# Prolonged mean response time in older adults with cardiovascular risk compared to healthy older adults

**DOI:** 10.1186/s13102-022-00565-4

**Published:** 2022-09-23

**Authors:** Kazuyuki Kominami, Masatoshi Akino

**Affiliations:** 1 Department of Rehabilitation, Sanseikai Kitano Hospital, 6-30, 1-chome, Kitano1-jyo, Kiyota-ku, 004–0861 Sapporo, Hokkaido Japan; 2 Department of Rehabilitation, Sapporo Kiyota Orthopedic Hospital, 1-50, 4-chome, Kiyota1-jyo, Kiyota-ku, 004-0841 Sapporo, Hokkaido Japan

**Keywords:** Mean response time; cardiopulmonary exercise testing, Exercise prescription, Exercise intensity, Older population

## Abstract

**Background:**

During incremental exercise (Inc-Ex), the mean response time (MRT) of oxygen uptake (V̇O_2_) represents the time delay before changes in muscle V̇O_2_ reflect at the mouth level. MRT calculation by linear regression or monoexponential (τ’) fitting of V̇O_2_ data are known to be highly variable, and a combination of incremental and constant load exercise (CL-Ex) is more reproducible.

**Methods:**

We evaluated MRT in older adults using linear regression and combination methods. We recruited 20 healthy adults (male: 9, 69.4 ± 6.8 years) and 10 cardiovascular risk subjects (male: 8, 73.0 ± 8.8 years). On day 1, they performed Inc-Ex using a 10W/min ramp protocol, for determination of the ventilatory anaerobic threshold (VAT) using the V-slope method. On day 2, they performed Inc-Ex to VAT exercise intensity and CL-Ex for 25min total. The MRT was calculated from the CL-Ex V̇O_2_ average and the time at equivalent V̇O_2_ in the Inc-Ex. We also assessed the amount of physical activity using the International Physical Activity Questionnaire short form (IPAQ-SF).

**Results:**

The MRT of healthy participants and those at cardiovascular risk were 49.2 ± 36.3 vs. 83.6 ± 45.4s (p = 0.033). Total physical activity in the IPAQ-SF was inversely correlated with MRT.

**Conclusion:**

The MRT was significantly prolonged in cardiovascular risk participants compared to healthy participants, possibly related to the amount of daily physical activity. Individual MRT may be useful for adjustment of exercise intensity, but this should also be based on daily physical activity and individual condition during exercise.

## Introduction

For accurate exercise prescription and cardiorespiratory health assessment, cardiopulmonary exercise testing (CPET) has become the gold standard protocol in research and clinical practice for quantifying main aerobic parameters (e.g., maximal V̇O_2_peak [peak oxygen uptake], ventilatory anaerobic threshold [VAT] and respiratory compensation point [RCP]) [1]. These incremental-derived parameters are then used as reference metrics to prescribe exercise intensity for individuals with cardiovascular disease and a variety of other medical conditions, including obesity and hypertension [2]. For example, exercise intensity in cardiac rehabilitation is often prescribed to be within 50–80% of VO_2_peak or 40–85% of VAT [3–6].

During incremental exercise, oxygen uptake (V̇O_2_) measured at the level of the mouth increases linearly with the power output after an initial time interval, the mean response time (MRT) [7], due to a delay in biological response. This includes both the transit delay for deoxygenated blood from exercising muscles to reach the lungs and the kinetic component of muscle V̇O_2_ adapting to the increased demand for adenosine triphosphate due to exercise (oxygen transfer capacity and mitochondrial function) [8–12].

MRT is generally calculated by monoexponential (τ’) fitting or linear regression of V̇O_2_ data. The time constant “τ” indicates 63.2% of the time it takes for oxygen uptake to reach a steady state during exercise at a constant load intensity [13, 14]. This time constant “τ” is prolonged in patients with heart failure [15]. The method of linear regression is defined as the time from a steady state of warm-up oxygen uptake to the beginning of incremental exercise load and the start of a gradual increase in oxygen uptake. This has been shown to be influenced by the intensity of warm-up and incremental exercise [16, 17]. Furthermore, both methods lack reproducibility [12, 18, 19]. The exercise intensity for exercise therapy in cardiac rehabilitation may also be corrected to account for this biological response delay (approximately 1min before that intensity) [20]. However, few reports have examined MRT to a given exercise intensity as a basis for exercise prescription with the 1-min pre-correction that is used as a rough approximation [13, 21].

Recently, a new method for obtaining reproducible MRT has been reported by Iannetta et al. [22]. This new method combining constant and incremental load exercise was able to reduce the effects of baseline before the incremental load and ΔVO_2_/Δpower output [22]. We hypothesized that this method could be used to calculate MRT in the older population and patients at cardiovascular risk and that patients at cardiovascular risk would have a prolonged MRT compared to healthy older participants. Therefore, the purpose of this study was to determine the extent of MRT, defined as the difference in response time between an incremental exercise and a constant load exercise, in older healthy participants and patients with cardiovascular risk.

## Methods

The study included a total of 30 participants, and all participants were between the ages of 60 and 80 years. We recruited 10 patients on medication for cardiovascular diseases (n = 5) or cardiovascular risk factors (n = 5) (risk group, age: 73.0 ± 8.8 years). Cardiovascular disease etiologies included post-coronary artery bypass graft surgery (n = 2), myocardial infarction (n = 1), and valvular heart disease (n = 2). Cardiovascular risk factors included hypertension (n = 10), impaired glucose tolerance or diabetes mellitus (n = 1), and hyperlipidemia (n = 6). Twenty healthy individuals matched for age (healthy group, age: 69.4 ± 6.8 years) were recruited for comparison (Table1). To estimate the daily activity levels of the participants, the International Physical Activity Questionnaire (IPAQ) short form was administered [23].

**Table 1:** Clinical characteristics of study participants

Characteristics		Healthy group [n = 20]	Patient group [n = 10]
Age	[years]	69.4 ± 6.8	73.0 ± 8.8
Sex		M:9, F:11	M:8, F:2
Height	[cm]	159.4 ± 5.9	164.7 ± 3.8
Body weight	[kg]	56.9 ± 8.3	67.1 ± 10.5
BMI		22.3 ± 2.2	24.8 ± 4.1
CTR	[%]		47.9 ± 4.7
BNP	[pg/dL]		73.9 ± 126.4
LAD	[mm]		36.3 ± 4.9
LVDd	[mm]		49.2 ± 5.9
LVDs	[mm]		29.5 ± 8.1
LVEF	[%]		68.1 ± 13.4
E/A ratio			1.24 ± 1.13
IPAQ-SF	[MET-min/week]	2082 ± 1857	3895 ± 4371
Comorbidity			
Hypertension	[n (%)]	0 (0)	10 (100)
Dyslipidemia	[n (%)]	0 (0)	6 (60)
impaired glucose tolerance	[n (%)]	0 (0)	1 (10)
Obesity	[n (%)]	2 (10)	4 (40)

Data are presented as mean ± S.D. Obesity is defined as BMI > 25kg/m^2^. Significant differences in clinical characteristics such as age, BMI, and physical activity (measured by the IPAQ-SF) were not observed between healthy and patient groups. CTR, cardio-thoracic ratio; BMI, body mass index; BNP, brain natriuretic peptide; LAD, left atrial diameter; LVDd, left ventricular diastolic diameter; LVDs, left ventricular systolic diameter; LVEF, left ventricular ejection fraction; E/A ratio, the peak early diastolic filling velocity/the peak atrial filling velocity ratio; IPAQ-SF, international physical activity questionnaire–short form; MET, metabolic equivalent.

Exclusion criteria included changes in medication within six months, infection within two weeks, chronic atrial fibrillation or flutter, permanent pacemaker, and presence of orthopedic conditions that rendered the individual unfit for exercise testing. In addition, we excluded participants who took warfarin, other anticoagulants, or metformin for diabetes.

Echocardiography and blood samples were measured prior to the day 1 incremental exercise testing. Left ventricular ejection fraction (LVEF) was obtained by Teichholz method. Brain natriuretic peptide (BNP) was determined by chemiluminescent enzyme immunoassay.

The cases and measurement records for this study were taken from participants in our previous article [24].

### Ethical considerations

The study was conducted in accordance with the principles outlined in the Declaration of Helsinki and was approved by the ethical committee of Sapporo Ryokuai Hospital (approval number: 19–1). Informed consent was obtained from all participants for their participation in the study and for the publication of this report.

### Exercise testing

**Fig. 1:**
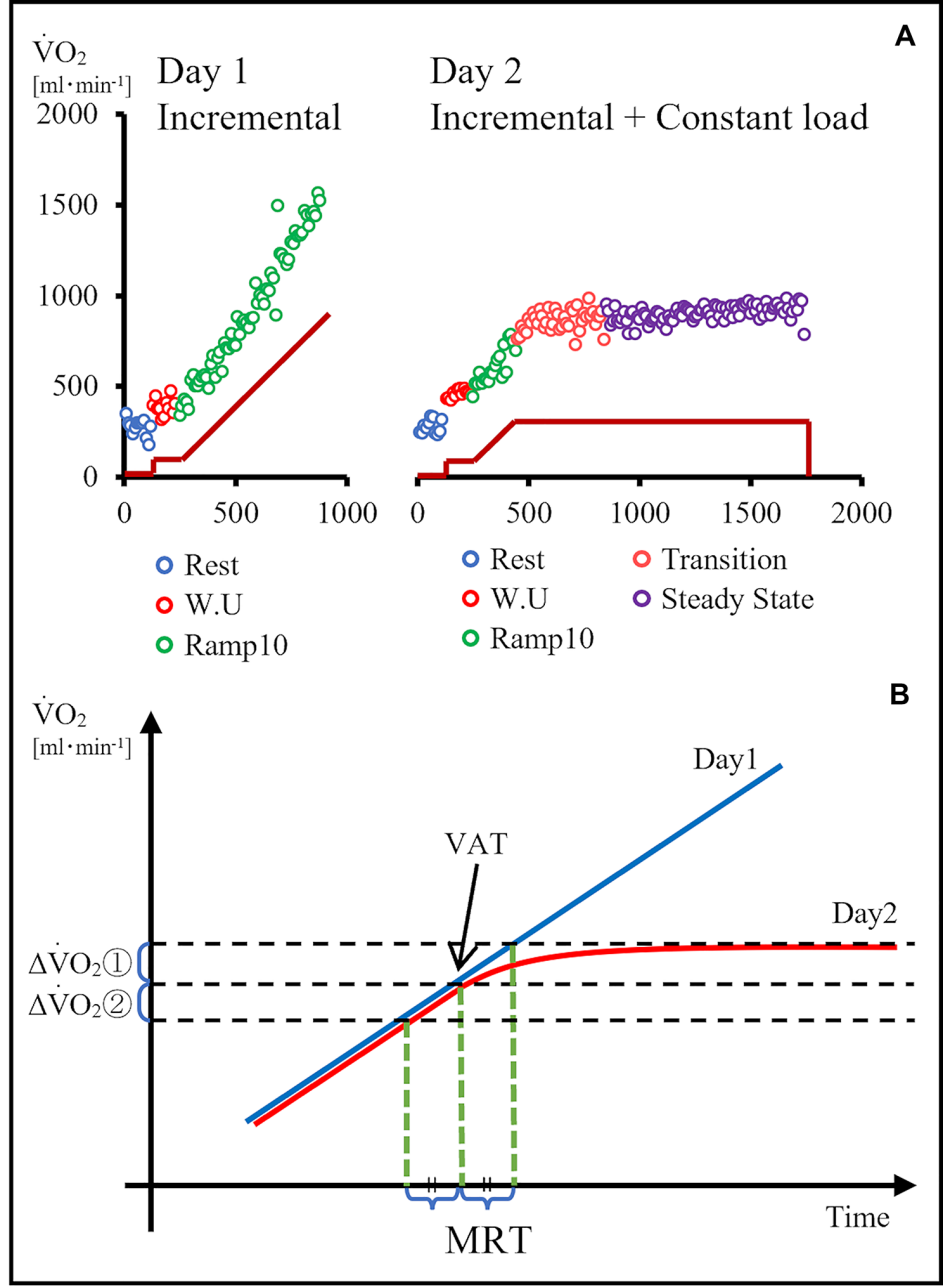
Diagram of Mean Response Time Calculation. (**A**) (upper panel) shows V̇O_2_ during incremental and constant exercise load in a sample case. Rest, warm-up, and a ramp protocol of 10W/min (Ramp10) on day 1 was followed on day 2 by constant load exercise for a total of 25min. (**B**) (lower panel) shows a diagram of the MRT calculation. The difference in oxygen uptake from VAT on day 1 to V̇O_2_ steady state (20–25min) on day 2 was defined as ΔV̇O_2_ ① (ΔV̇O_2_ after VAT). The difference in oxygen uptake from the day 1 VAT to the earlier MRT was defined as ΔV̇O_2_ ② (ΔV̇O_2_ before VAT). VAT – ventilatory anaerobic threshold; V̇O_2_ – oxygen uptake

CPET was performed using a stationary bicycle (Strength Ergo 8; Mitsubishi Electric Engineering, Tokyo, Japan) and a breath-by-breath gas analyzer (AE-300S; Minato Ikagaku Co., Tokyo, Japan). Exercise tests were conducted on two separate days (mean interval between the 1st- and 2nd-day tests: 4.1 ± 2.3 days). On day 1, symptomatic maximal exercise was performed using a ramp protocol of 10W/min (Inc-Ex) for VAT determination. On day 2, Inc-Ex was performed using a ramp protocol of 10W/min up to the VAT point, after which a constant load at the VAT level work rate was initiated and maintained for a total exercise duration of approximately 25min (Fig.1). Before the experiment, the total duration of the exercise (Inc-Ex + CL-Ex) on day 2 was planned to be 25min for each participant. The duration of Inc-Ex varied among participants due to different VAT levels. Consequently, the mean Inc-Ex duration was 3.2 ± 1.1min and the mean CL-Ex duration was 21.8 ± 1.1min. Thus, all graphs, tables, and text denoting 25min of CL-Ex represent approximately 22min of CL-Ex. Warm-up exercises were performed for 2min at 10W. We used 10-s average data for all analyses. This exercise testing protocol has been published previously [24].

### Ventilatory anaerobic threshold

We determined the VAT during Inc-Ex testing on day 1 to determine the CL-Ex work rate on day 2. The VAT was visually determined using the modified V-slope method as described by Sue et al. [25], which is a modification of the method described by Beaver et al. [26]. The details of this method have been published previously [27, 28].In summary, this V-slope method involves drawing a line through the data points parallel to the respiratory exchange ratio (RER) = 1 diagonal, which is referred to as the pre-VAT baseline (S1). The point at which the data begin to deflect toward the left is selected as the VAT. The data points preceding the parallel line were disregarded. A line drawn parallel to the RER = 1 diagonal signifies a change of 1.0 in the rate of ΔV̇CO_2_/ΔV̇O_2_. Therefore, the point at which this index begins to increase above 1.0 is the VAT deflection point [27, 28]. Previous studies included actual readings of the VAT for each analyzed case. We used this approach in our study to identify the VAT.

### Calculation of MRT (Fig.1)

We calculated the MRT from the oxygen uptake of the incremental and constant load exercises (Inc-Ex and CL-Ex, respectively) following the method of Iannetta et al. (MRT1) [22]. We calculated the oxygen uptake for 20–25min during CL-Ex and defined MRT1 as the difference between the time to achieve this same oxygen uptake and the time to the VAT during Inc-Ex. The difference in oxygen uptake before and after MRT1 based on VAT was also calculated during incremental exercise testing. Detailed exercise testing results such as V̇O_2_ and HR have been published previously [24].

In addition, we also calculated the method by linear regression (MRT2). Referring to previous studies, MRT2 was calculated from the intersection of oxygen uptake at warm-up and the linear regression equation of oxygen uptake from the start of increased oxygen uptake to VAT during incremental exercise testing [16, 17].

### Statistical analysis

Data are presented as mean ± SD and 95% confidence interval. All data were assessed for normality by the Shapiro-Wilk test before analysis. Within group comparisons, such as MRT1 and MRT2, and ΔV̇O_2_ before and after VAT, were made using a paired t-test. Comparisons between groups in the healthy and at-risk groups were performed using the student t-test. Correlation of total metabolic equivalents (METs) in IPAQ-short form and MRT1 was performed using Pearson’s correlation. P < 0.05 was considered to indicate statistical significance.

Statistical analyses were performed with Statistics for Excel 2012 (Social Survey Research Information Co., Tokyo, Japan).

## Results

The clinical characteristics of the participants are summarized in Table1. Compared to the healthy group, the cardiovascular risk group had a higher proportion of men, resulting in significantly higher height and body weight (p = 0.016, p = 0.007, respectively). The total MET minutes per week according to the IPAQ-SF was not significantly different between the healthy and risk groups (p = 0.119). This result is similar to the average of a broader healthy Japanese population in the same age range [29]. MRT1 could be calculated in all cases.

### MRT - combined calculation of incremental and constant load exercise

**Fig. 2:**
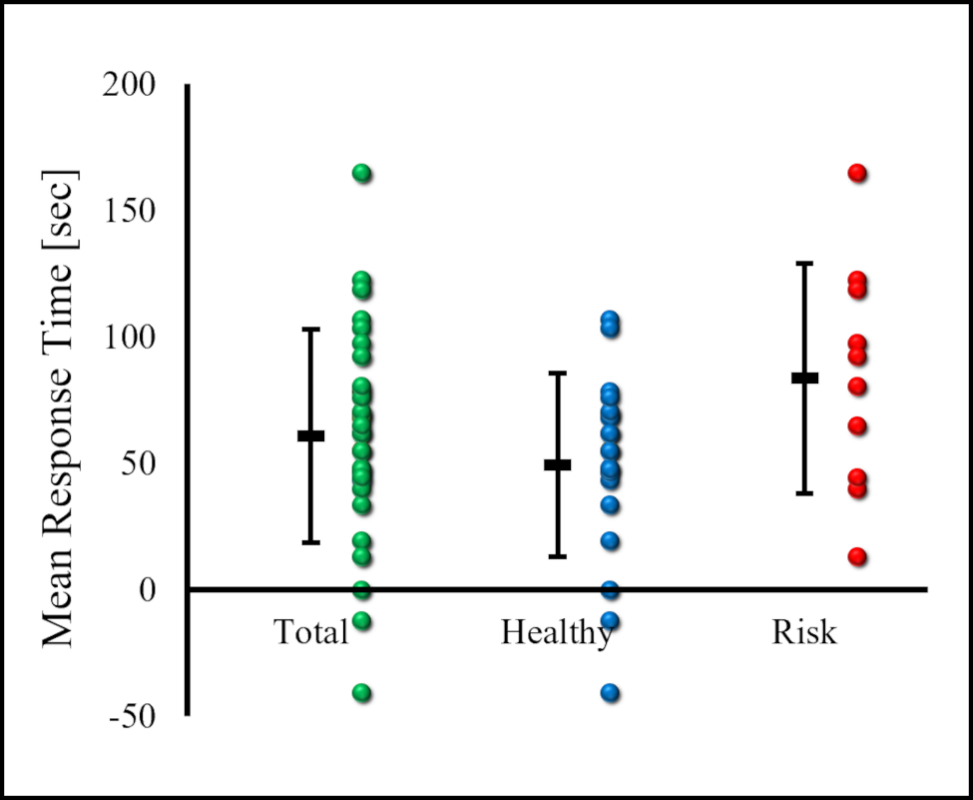
Mean Response Time. The mean response time (MRT) for each group is shown. Green markers indicate individual mean response times for all cases. Blue markers indicate individual mean response times for the healthy group and red markers indicate individual mean response times for the cardiovascular risk group. Patients with cardiovascular risk had significantly prolonged MRT compared to healthy adults

MRT1 was 60.7 ± 42.1 sec(95% CI: 45.6–75.8) in all participants, 49.2 ± 36.3 sec(95% CI: 33.3–65.1) in the healthy group, and 83.6 ± 45.4 sec(95% CI: 55.4–111.7) in the risk group. The risk group had a significantly prolonged MRT compared with the healthy group (p = 0.033) (Fig.2).

### Differences in MRT by calculation method

In the healthy group, MRT1 and MRT2 were not significantly different and not correlated (49.2 ± 36.3 vs. 46.8 ± 16.1sec, p = 0.797, r=−0.149, p = 0.681). On the other hand, in the risk group, MRT2 was significantly lower than MRT1, but no correlation was found (83.6 ± 45.4 vs. 26.4 ± 23.9sec, p = 0.010, r = 0.269, p = 0.451).

### Differences in ΔV̇O_2_ before and after VAT

**Fig. 3:**
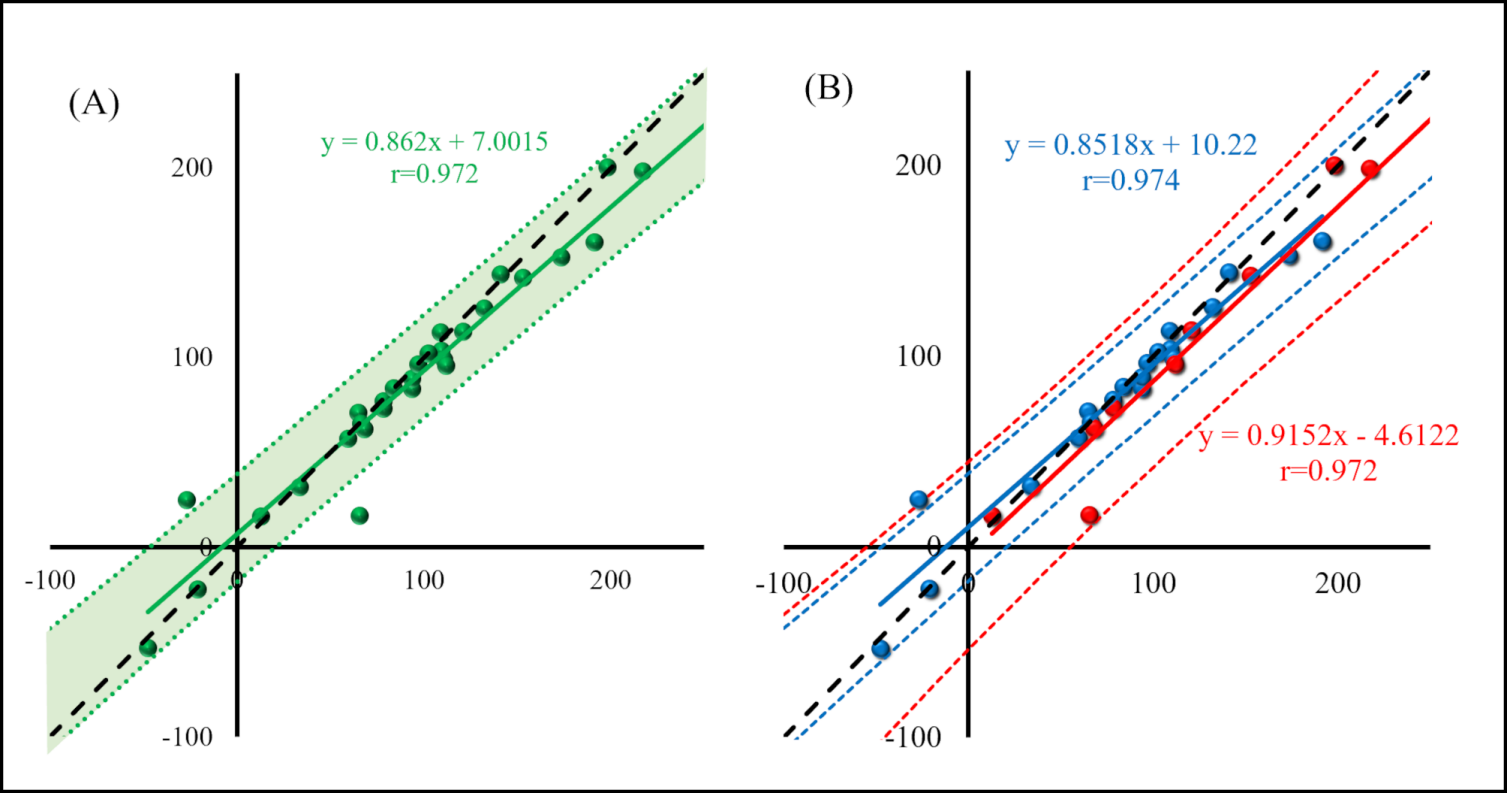
ΔV̇O_2_ before and after VAT. The left figure (**A**) shows all cases (green) and the right figure (**B**) shows pre- and post-VAT ΔV̇O_2_ for each group (healthy group: blue; risk group: red). The x-axis shows ΔV̇O_2_ after VAT and the y-axis shows ΔV̇O_2_ before VAT. Dotted lines indicate 95% confidence intervals for each group in their associated color. ΔV̇O_2_ showed a significant correlation in both groups. Patients with cardiovascular risk had significantly higher ΔV̇O_2_ compared to healthy adults. VAT – ventilatory anaerobic threshold. V̇O_2_ – oxygen uptake

ΔV̇O_2_ before and after MRT with respect to VAT showed a significant correlation, but was significantly higher in the risk group (all patients: 91.3 ± 62.2 vs. 97.8 ± 70.1 ml‣min^-1^, p = 0.051, healthy group: 80.2 ± 53.9 vs. 82.2 ± 61.7ml‣min^-1^, p = 0.573, risk group. 113.5 ± 74.2 vs. 129.0 ± 78.8ml‣min^-1^, p = 0.028, Fig.3).

### Relationship between daily physical activity and MRT

**Fig. 4:**
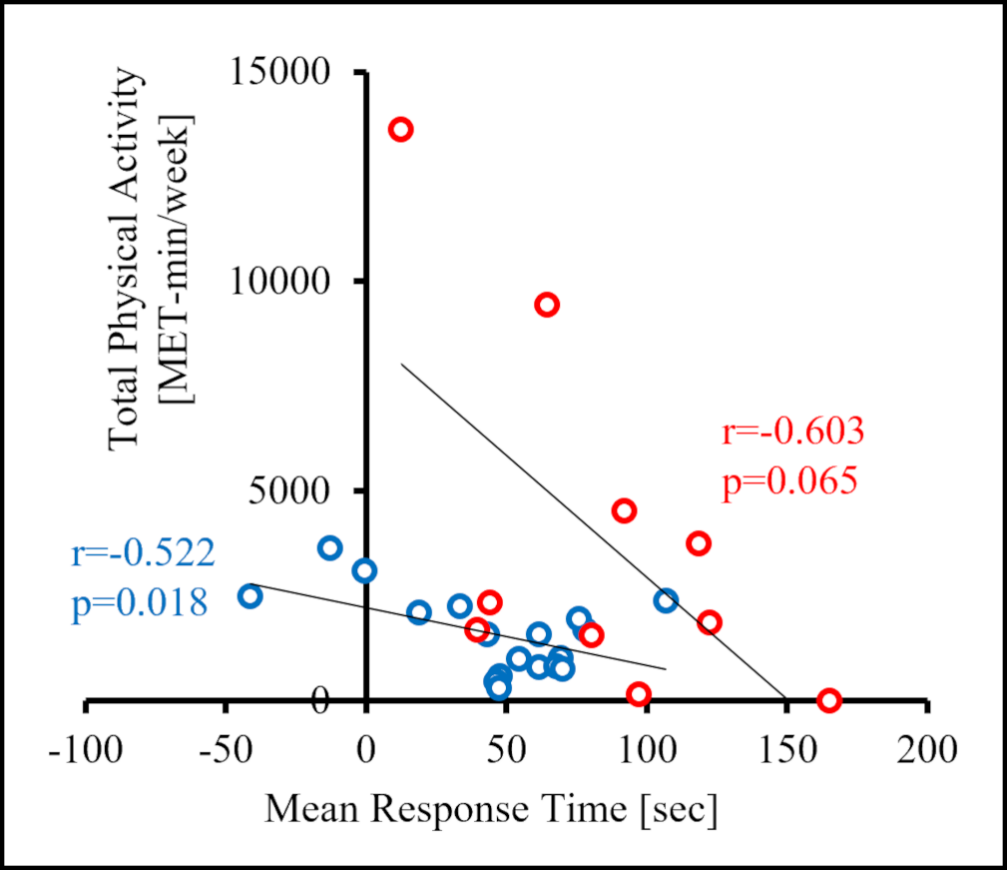
Relationship between mean response time and physical activity. Physical activity (in metabolic equivalents, METs) was calculated using the responses from the International Physical Activity Questionnaire short form (IPAQ-SF). Mean response time (MRT) tended to shorten in both groups (healthy group: blue; risk group: red) as activity level increased

Daily physical activity and MRT showed a negative correlation (healthy group: r=−0.522, p = 0.018, risk group: r=−0.603, p = 0.065). In both groups, the higher the daily physical activity level, the shorter the MRT tended to be (Fig.4).

## Discussion

Quantification of the MRT is paramount for assigning the correct load to the VAT [13, 21]. This study examined MRT, calculated using a method recently developed by Iannetta et al. [22], in a cardiovascular disease population and healthy age-matched controls. Furthermore, we compared this method against other methods that had been traditionally used in research settings (i.e., back extrapolation linear approach). The results showed that the MRT was significantly prolonged in cardiovascular disease patients when compared to controls individuals. Furthermore, there was no correlation between the MRT quantified with the novel method proposed by Iannetta et al. [22] and the back extrapolation linear approach. This means that for greater accuracy, MRT should be calculated using this novel method.

The results of this study showed that older adults with cardiovascular risk had a prolonged MRT compared to healthy older adults. Compared to previous studies, MRT was also prolonged in healthy young adults and athletes [9]. MRT may be prolonged due to multiple factors.

MRT is related to circulatory delay, pulmonary gas exchange and the oxygen uptake response of exercising muscle and increased ATP demand due to incremental exercise [22, 30]. Adequate blood flow to exercising muscles may also be relevant for MRT [8, 10]. Cardiac dysfunction such as hypertensive heart disease [31, 32], right ventricle heart failure [14, 33], left ventricular diastolic dysfunction [34–36], atherosclerotic diseases such as diabetes [37, 38], decreased physical activity [39], and age-related changes [39–41] are believed to produce a delayed or decreased cardiovascular response during exercise. Furthermore, it is reported that in pulmonary diseases, such as COPD, dynamic lung hyperinflation and pulmonary gas exchange limitation may occur [30].

In the present study, MRT was significantly prolonged in the risk group with comorbidities known to produce atherosclerosis, although there was no apparent decline in cardiac function. In addition, physical inactivity [42, 43], age-related changes [44], and comorbidities [45, 46] are believed to decrease mitochondrial function and ATP synthesis capacity. In this study, physical inactivity was shown to be associated with prolonged MRT. Although this study was not able to sufficiently investigate the possibility, it cannot be ruled out that the patients may have had coexisting pulmonary diseases, such as COPD. Altogether, these factors may have contributed to a prolonged MRT in patients at cardiovascular risk compared to healthy adults.

We used two methods to calculate MRT in this study: the method used in previous studies, which is based on a combination of incremental and constant load exercise, and a method based on a linear regression equation. In previous studies, the calculation of MRT by the linear regression equation was related to the intensity of constant load before the start of the incremental exercise (warming up intensity) and the intensity of ramp exercise [16, 17].

In this study, the ramp loading intensity was 10W/min in all cases, which was similar to the intensity of ramp exercise shown in previous studies. Conversely, although all cases of constant load exercise were standardized at 10W, the warm-up period was 2min, which may have been too short for oxygen uptake to reach a steady state. This may have affected the MRT calculated using the linear regression equation and its correlation with the MRT calculated by the combination method.

ΔV̇O_2_ was significantly higher in the cardiovascular risk group. The risk group had a higher initial ΔV̇O_2,_ which may be related to the prolonged MRT. ΔV̇O_2_ before and after VAT was correlated very strongly in both groups and was almost the same within the groups. However, the risk group was significantly higher, albeit slightly. The degree of increase in oxygen uptake in response to incremental exercise intensity depends on the setting of the individual exercise intensity (the increase in oxygen uptake slows down with increasing exercise intensity when the incremental exercise intensity is high) [47]. Additionally, the slow component of oxygen uptake increases with exercise intensity greater than VAT [48, 49]. In the present study, MRT was prolonged and ΔV̇O_2_ after VAT was greater in the risk group, suggesting that the slow component may have had an effect. This may also be related to the amount of physical activity and other factors which interact with prolonged MRT [50].

Correction of exercise prescription is recommended to account for a delayed biological response and tailor to the individual [3, 51]. The results of this study showed that the average MRT in the cardiovascular risk group was actually prolonged beyond 1min, which would be easy to recommend as a simple correction. However, the range of MRT is large, so this may not be directly accurate without individual testing. As reported in our previous studies, oxygen uptake at constant load exercise in VAT intensity was around 60% V̇O_2_ peak, well within the range of some guidelines [6, 52, 53]. Therefore, it would not be easy to overload beyond VAT. Based on the results of this study, when setting exercise intensity for exercise therapy, it is recommended that MRT should be calculated using this novel method for greater accuracy.

### Limitations

This study had some limitations. First, although it was clear that MRT was prolonged in patients with cardiovascular risk, the overall number of cases, especially in the risk group, was small, and disease specificity due to comorbidities was unclear. It is uncertain to what extent comorbidities such as hypertension and diabetes would alter MRT.

Second, the study population was not composed of patients with myocardial infarction or heart failure who were indicated for cardiac rehabilitation. MRT may be further prolonged in conditions that cause circulatory delay.

In summary, older participants with cardiovascular risk had a prolonged mean MRT compared to healthy older participants. This MRT prolongation may be linked to the impaired oxygen cascade in patients with cardiovascular disease. When setting exercise intensity for exercise therapy, we recommend that, for greater accuracy, the MRT should be calculated using this novel method.

## Data Availability

The dataset used in the current study is available from the corresponding author on request.

## Declarations

### Author details

1 Department of Rehabilitation, Sanseikai,6-30, 1-chome,Kitano1-jyo, Kiyota-ku,004–0861, Kitano Hospital, Sapporo, Hokkaido, Japan.

2 Department of Rehabilitation, Sapporo Kiyota Orthopedic Hospital, Sapporo, Hokkaido, Japan

## Abbreviations

GET: gas exchange threshold.
VAT: ventilatory anaerobic threshold.
Inc-Ex: incremental exercise.
CL-Ex: constant load exercise.
CPET: Cardiopulmonary exercise testing.
V̇O_2_: Oxygen uptake.
V̇CO_2_: carbon dioxide production.
RER: respiratory exchange ratio.
MRT: mean response time.
IPAQ: International Physical Activity Questionnaire.
LVEF: Left ventricular ejection fraction.
BNP: Brain natriuretic peptide.
ANOVA: A repeated one-way analysis of variance.
